# Effect of bismuth oxide nanoparticle on the electromagnetic interference shielding and thermal stability of industrial waste based-geopolymer composites

**DOI:** 10.1038/s41598-023-27623-2

**Published:** 2023-01-31

**Authors:** Christian V. Maestre, Gil Nonato Santos

**Affiliations:** Department of Physics, De La Salle University, Manila, Philippines

**Keywords:** Composites, Nanoparticles, Mechanical properties, Characterization and analytical techniques

## Abstract

Gold mine tailings, fly ash, and bagasse ash has been repurposed to produce geopolymer (GP) with enhanced electromagnetic interference shielding efficiency (EMI-SE) and high thermal property. GP has low shielding efficiency compared to concrete. Due to this, an appropriate filler must be incorporated into its matrix to enhance its EMI-SE. For this study, bismuth oxide nanomaterial (BiNP) was utilized as the additive filler. The percent content of BiNP was varied to evaluate its influence on the EMI-SE of GP. Morphology shows that Bi_2_O_3_ was embedded in the matrix of GP, and no new aluminu-phyllosilicate minerals were formed. This indicates that some minerals acted only as internal fillers in the matrix. Compressive strength shows synthesized GP composites were more than 20 MPa, with neat GP reaching the maximum strength. Moreover, the EMI-SE of neat GP was 21.2 dB for 20–4500 MHz range. This indicates that GP alone has sufficient characteristics to attenuate EMI radiation. Addition of 5%, 10% and 15% weight of BiNP improves EMI-SE by 4–10%, with 5% BiNP shown to be the optimum ratio. Lastly, the addition of BiNP improves the thermal stability of GP. This study shows that GP incorporated with Bi_2_O_3_ can be recommended for small-scale construction and small residential building.

## Introduction

The development of electronic devices and large equipment has benefited human society in recent years. However, it also raised a lot of concern due to the unprecedented rise of electromagnetic radiation (EMR) pollution, which may affect human health^1,2^. EMR source signal has also caused issues in various industries, such as the aviation and medical sector. It also triggered malfunctions and deterioration of electronic devices and could be used as military weapons (EM pulse) to neutralize networks of an enemy^3–5^. Due to this, research with regard to fabricating materials with significant EM shielding properties is required to reduce potential human exposure to EM radiation and protect sensitive electronic devices.

Most studies directed into EMI shielding material are aimed at lightweight materials such as coating and thin panels, usually fabricated based on foams and polymers containing carbon-based materials and conductive and magnetic absorbers. Carbon-based materials have been extensively used as effective absorbing materials, such as graphene, graphite, carbon fibers, and nano forms of carbons such as nanotubes (CNT’s) and nanorods. However, large-scale EMR protection, such as in construction applications with enhanced shielding properties to restrict EMR admittance, would be a disadvantage for these polymer-based materials due to their poor mechanical properties and chemical stability. In recent years, attention has been targeted to developing a construction material that would act as a shield against EM radiation and would not require additional carbon-based filler. Much of this attention has been centered on Ordinary portland cement (OPC) due to its intrinsic water content, high-density property, low cost, and ease of handling in small or large-scale applications. However, its production is responsible for a large amount of greenhouse gases, which account for 5–7% of the total anthropogenic CO2 emission^6^. This led many researchers globally to pursue alternative and lower carbon footprint materials.

Furthermore, in a low-resource environment where OPC and its constituent components are difficult to procure, an alternative to concrete is necessary: indigenous or locally available source materials. One material that has become widely popular in the last decade that could potentially replace OPC-based concretes is geopolymer. This material is an alkali-activated aluminosilicate binder that takes natural and/or industrial wastes as the primary raw material.

Geopolymers are synthesized via the geopolymerization process, which is done by transforming an aluminosilicate material into an amorphous phase and covalently bonded three-dimensional network bonds of –Si–O–Al–O– material in an ambient or high-temperature condition^7^. This process involves the dissolution of aluminosilicate material in a concentrated alkaline solution, followed by the reorganization and diffusion of dissolved ions with the formation of small, coagulated structures and, eventually the polycondensation of soluble species to form hydrated products. The majority of the source materials used in synthesizing geopolymer are industrial waste products from mining and metallurgical manufacture, which contains a rich amount of amorphous silica and aluminum. Hence, this material could reduce greenhouse gas emissions by 80% during production compared to OPC^8^. Furthermore, developing geopolymers with significant EM wave absorption, shielding properties, and thermal properties is essential to realize the overall goal of green buildings.

The structure of geopolymer consists of a 3-dimensional matrix, which limits the presence and movements of free-electron. Furthermore, the electrical conductivity of this material is directly related to the alkali ions present in its framework; however, the mobility of such ions is limited. This makes geopolymer have low electrical conductivity, and its EM wave absorption and shielding performance are low compared to OPC. Therefore, it is practical to enhance its effectiveness by introducing a suitable admixture.

Several research has incorporated various conductive fillings and loading to cement-based, such as carbon nanotubes with controllable silica shells^9^, graphene-coated CNT’s^10,11^, nickel fiber^12^, stainless-steel furnace dust^13^ and metallic nanoparticles such as cobalt, nickel, and iron oxide^14^. One potential metallic nanoparticle that could be used as a conductive admixture is Bismuth Oxide (Bi_2_O_3_), due to its good absorbing properties^15^. When Bi_2_O_3_ is added as a filler or dopant, the uniformity and density of the material will improve and, consequently, its crystallinity. Furthermore, it will reduce the porosity, improve the saturation magnetization and real permeability, and increase the dielectric constant^16^. Incorporating Bi_2_O_3_ nanoparticles could enhance the EMI shielding effectiveness (EMI-SE) of geopolymer; however, there are no existing studies concerning this matter. Furthermore, no work has yet been accomplished to evaluate the EMI shielding performance of geopolymer composites integrated with Bi_2_O_3_ nanoparticles.

The purpose of this study is to explore the influence of the content and proportion of Bi_2_O_3_ nanoparticles on the electromagnetic interference shielding effectiveness (EMI-SE) of geopolymer composites using industrial wastes such as Au mine tailings (AMT), coal fly ash (CFA), and sugar cane bagasse ash (BA) as aluminosilicate source materials. Furthermore, this investigation intends to provide proof-of-concept and demonstrates the potential of utilizing eco-friendly and low-cost geopolymer composites as an innovative EMR shielding material for construction applications.

## Materials and methods

### Collection and preparation of materials

**Figure 1:**
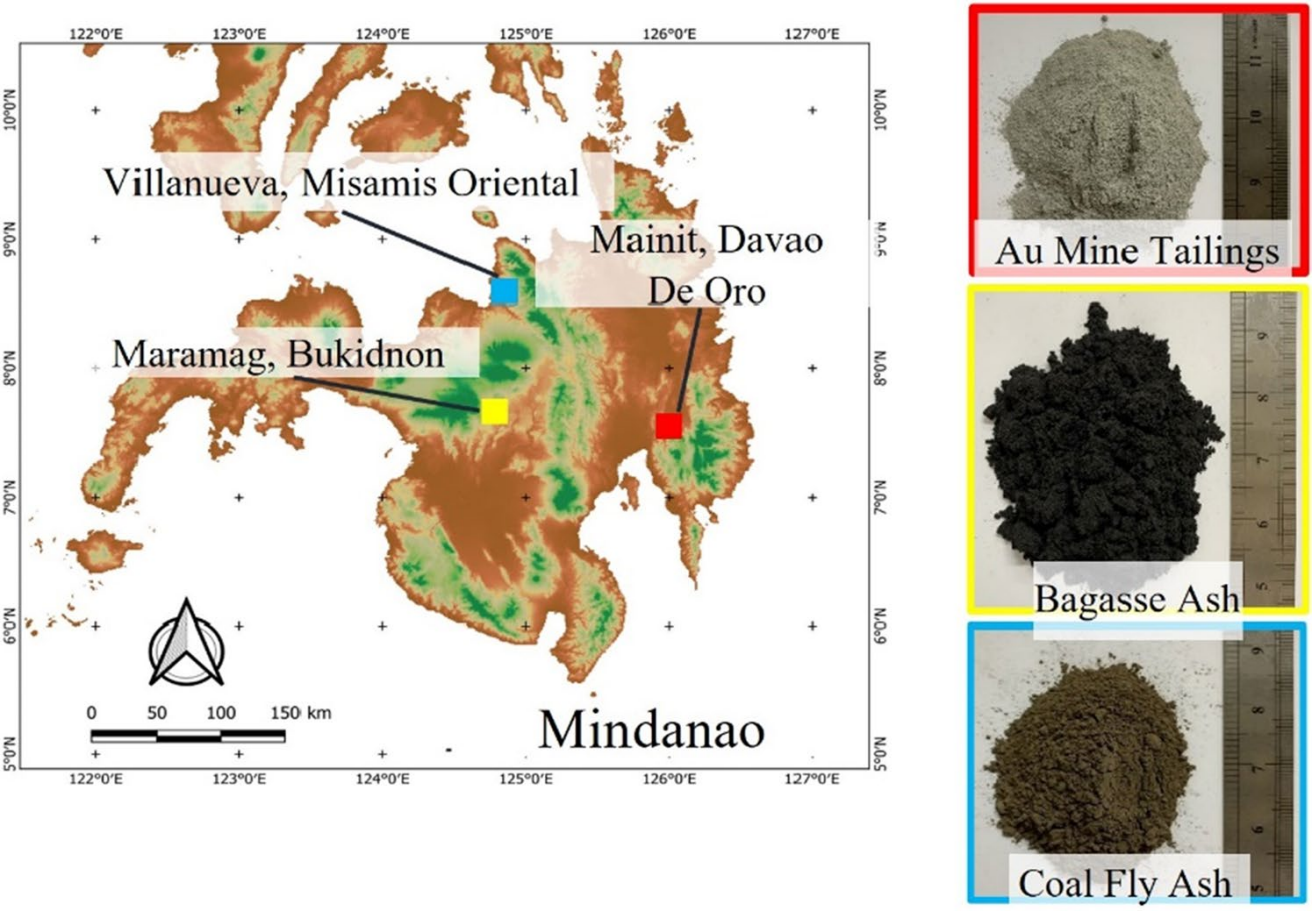
Map of Mindanao and the locations of the impounding facilities (source: QGIS 3.22.12, (https://www.qgis.org/en/site/index.html); and the as-collected raw samples.

Collection and preservation of raw industrial waste were carried out following the ASTM-D4220 (*Standard practices for preserving and transporting soil samples*)^17^. Artisanal and small-scale gold (Au) mine tailings were collected in an open “dampacan”, or impounding facilities of a carbon-in-pulp (CIP) plant located in Mainit, Davao de Oro (formerly Compostella Valley). Coal Fly Ash (FA) and Sugar Cane Bagasse ash (BA) were obtained in a coal power plant in Villanueva, Misamis Oriental, and a sugar refining manufacturing company in Maramag, Bukidnon, Philippines, respectively. Figure 1 shows the location of the sites, obtained from QGIS 3.22.12, an open-source data of geospatial information, and the as-collected raw samples. All-raw materials were air dried and sieved in a 2-mm aperture screen prior to preliminary analysis and geopolymerization procedure. Laboratory-grade sodium hydroxide pellets (99% purity) and hydrochloric acid (HCl) (36% v/v) were supplied by Merck. While bismuth (III) nitrate pentahydrate (Bi (NO_3_)·5H_2_O), sodium sulfate (Na_2_SO_4_), and ethanol (C_2_H_6_O) were obtained.

### Preparation of bismuth oxide nanoparticles

**Figure 2:**
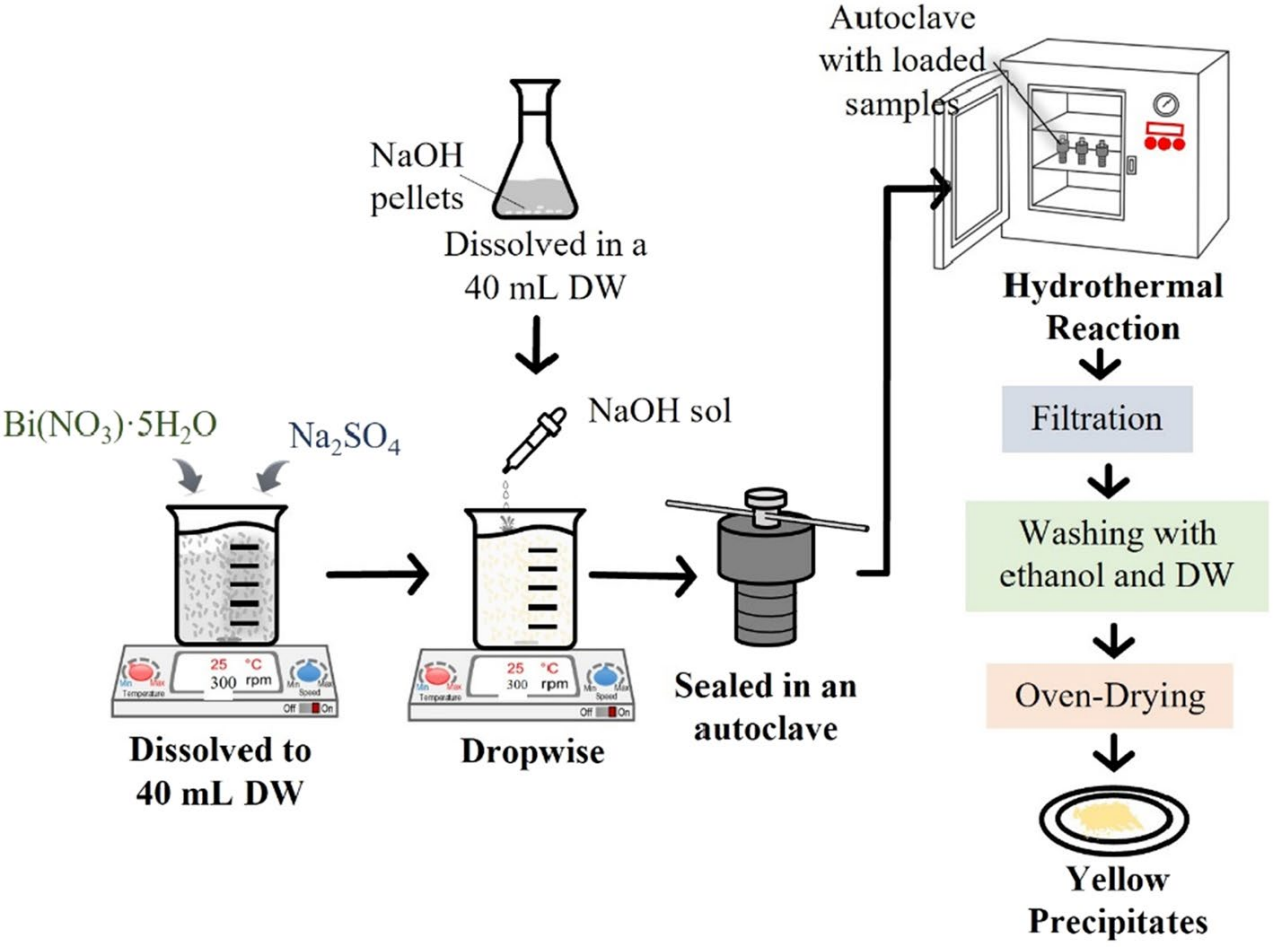
Flow chart of the hydrothermal synthesis of Bi_2_O_3_ NP.

Synthesis of Bi_2_O_3_ nanoparticles (Bi_2_O_3_ NP) were carried out via hydrothermal technique. For this method, 2.0 mmol of Bi(NO_3_)·5H_2_O and 3.0 mmol of Na_2_SO_4_ was mixed and dissolved in 40 mL of deionized water. The suspension was mechanically stirred for 2 h in a magnetic stirrer at moderate speed and set at ambient temperature (28–32 °C). Subsequently, 18.0 mmol of NaOH solution was added dropwise in the suspension and kept stirring for another 2 h. Afterward, the suspension was transferred, sealed in an autoclave, and subjected to a hydrothermal reaction at 65 °C temperature for 10 min. Then, the suspension was separated using a filtration and washed thoroughly with ethanol and distilled water. Finally, the Bi_2_O_3_ NP was obtained by oven drying the washed precipitates at 100 °C temperature for 2 h. Figure 2 shows a brief schematic illustration of the preparation of Bi_2_O_3_ NP.

### Preparation of powder activator and geopolymer composites

Powder-based activator used as an activating agent in geopolymerization were fabricated according to the modified method presented by Aseniero et al. (2019)^18^. In this study, BA was used as the precursor. As-collected BA was washed with 0.1 M HCl solution with 2.5 w/w acids to solid ratio and mechanically stirred for 20 min. It was then left for 12 h to settle. Afterward, the precursor was separated via decantation and filtration, and thoroughly rinsed with ethanol and water. The washed precursor was oven dried at 250 °C temperature for 8 h. Subsequently, the oven-dried precursor was mixed with 5 M NaOH solution at a 0.5 (w/w) mix ratio and mechanically stirred. Finally, the powder-based activator was obtained by drying the paste at 100 °C temperature for 8 h.

Geopolymer composites containing various amounts of Bi_2_O_3_ NP (5, 10, and 15% w/w) were fabricated. Table 1 shows the geopolymer mixing design. All samples were mixed at an appropriate mixing ratio to obtain a one-part mix of geopolymer cement. Twenty (20) % of water equivalent to the total weight of the geopolymer cement was added. Then, it was mixed in a rotary mixer for 15 min to form a slurry. Afterward, the slurry was transferred into a mold and left to settle for 24 h, set at ambient condition. Subsequently, the geopolymer composites were demolded and cured at 65 °C for 8 h.

**Table 1:** Geopolymer mixing design (% weight).

Sample ID	AMT	SA	CFA	BiNP
GP-1	35	50	15	15
GP-2				10
GP-3				5
GP-4				0

*AMT* mine tailings, *SA* solid activator, *CFA* fly ash, *BiNP* bismuth nanoparticles.

### Materials characterization

Surface Morphology and the dispersion of Bi_2_O_3_ nanoparticles in the geopolymer (GP) matrix were determined by field emission scanning electron microscopy (FESEM, Phenom XL) coupled with elemental dispersive x-ray (EDX) for chemical analysis. Prior to FESEM analysis, specimens were gold coated using JEOL JFC-1200 Fine coater to induce conductivity on their surface. The GP samples were molded to a standard cylindrical size to fit the waveguide test set-up with a thickness of 10 mm. A 50-mm cube was used to shape the geopolymer for the unconfined compression strength test. Unconfined Compressive Strength (UCS) was conducted on three-replicate cubic per sample ID based on the ASTM C109/C109M (ASTM, 2016) using a universal testing machine (UTM). The samples were tested for UCS after 28 days of curing. Thermal properties through thermogravimetric-differential thermal analysis (TG–DTA) were carried out by heating the sample in a nitrogen atmosphere at 50 mL/min up to 1000 °C using a simultaneous thermal analyzer. The equipment used was a Perkin Elmer STA 6000 with heating from 30 to 950 °C.

### Electromagnetic interference shielding efficiency set-up

**Figure 3:**
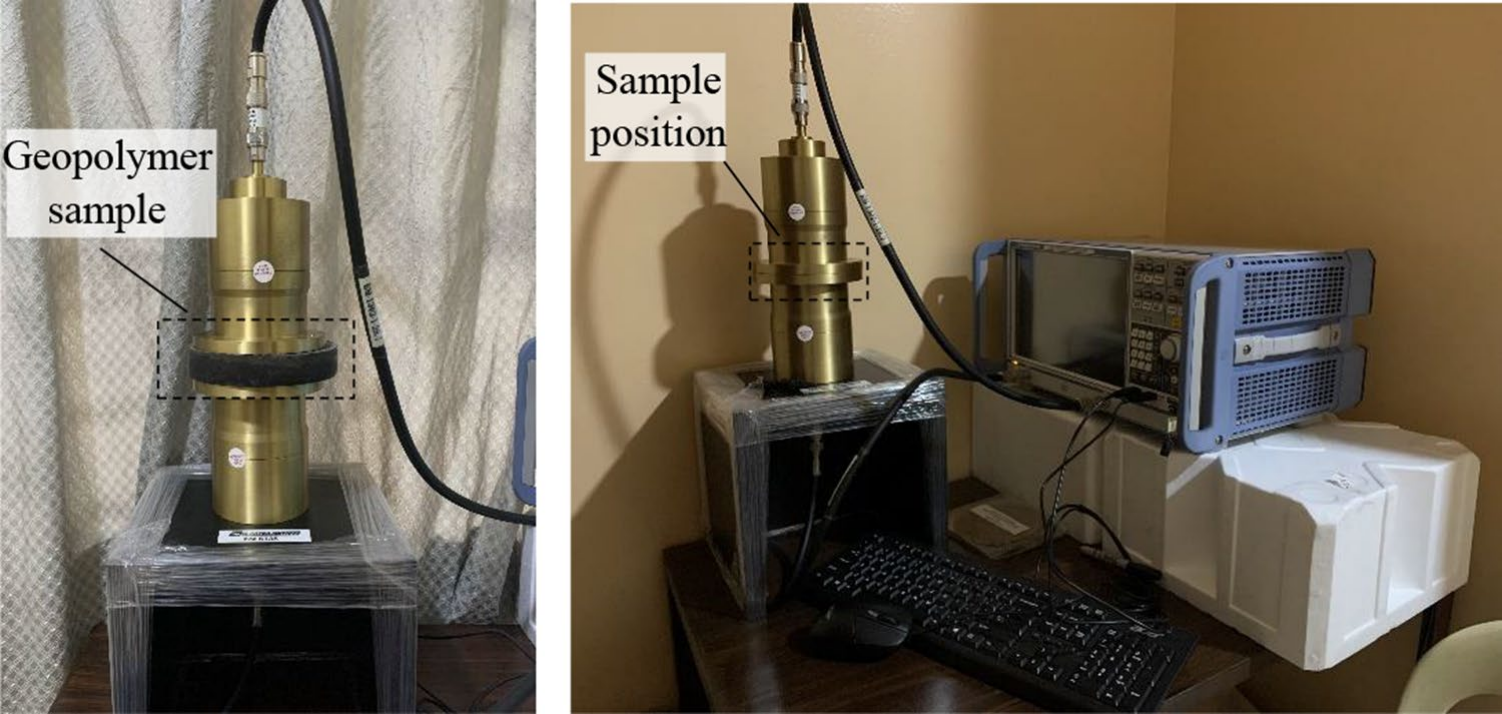
EMI-SE experimental set-up.

The Electromagnetic interference shielding efficiency (EMI-SE) test of the synthesized geopolymer was measured following ASTM D4935-18 standard (*Standard Test Method for Measuring the Electromagnetic Shielding Effectiveness of Planar Materials*)^19^ using a R&S®ZNL Vector Network Analyzer in the frequency ranges from 20 MHz < f < 4500 MHz. as shown in Fig. 3. The results of the scattering parameters (S11, S12, S22, and S21) were determined using the waveguide method, where the samples were placed in the coaxial specimen holder. EMI-SE of the samples was investigated by two consecutive transmission measurement bell-shaped adapters with and without a mounted specimen. S11 and S22 are designated as the reflection parameters, while S12 and S21 are the transmission and were quantified by

1 $$R = \left| {S_{{{11}}} } \right|^{{2}} = \left| {S_{{{22}}} } \right|^{{2}} ,{\text{ and}}\,\,\,T = \left| {S_{{{21}}} } \right|^{{2}} = \left| {S_{{{12}}} } \right|^{{2}} ,$$

where R is the reflection phenomena and T is the transmission phenomena. Furthermore, the value of absorption parameters was obtained using the equation.

2 $$A = { 1 } - \, \left( {T + R} \right)$$

Now, using these parameters, ${SE}_{A}, {SE}_{R}$, and ${SE}_{MR}$ were quantified by.

3 $${SE}_{R}\left(dB\right)=10{log}_{10}\left(\frac{1}{1-R}\right)$$

4 $${SE}_{A}\left(dB\right)=10{log}_{10}\left(\frac{1-R}{T}\right)$$

5 $${SE}_{MR}\left(dB\right)=20{log}_{10}\left(1-{R}^{2}\left({10}^{-\frac{{SE}_{A}}{10}}\right)\right)$$

The total shielding effectiveness was then calculated.

6 $$SE_{R} \left( {dB} \right) = SE_{R} + SE_{A} + SE_{MR}$$

## Results and discussion

### Properties of industrial raw materials

**Figure 4:**
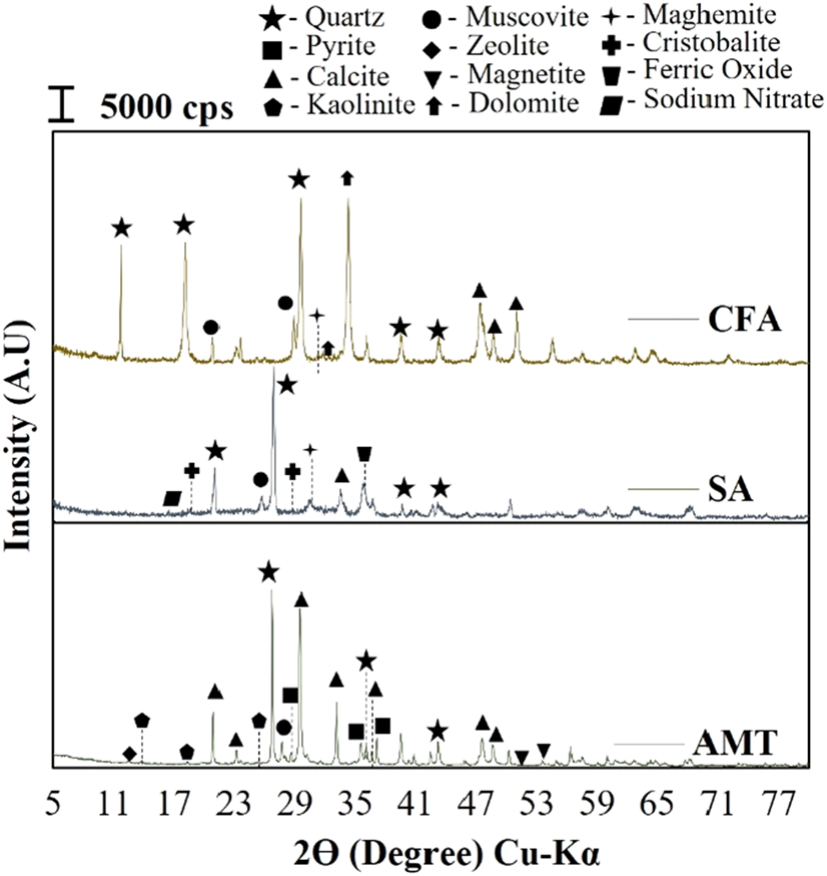
XRD pattern of the industrial waste materials.

The XRD patterns of the industrial raw materials used in this study are shown in Fig. 4. The minerals present in the as-collected Au Mine Tailings (AMT) are quartz (SiO_2_), pyrite (FeS_2_), calcite (CaCO_3_), kaolinite (Al_2_Si_2_O_5_(OH)_4_), zeolite (Na_2_Al_2_Si_2_O_8_), muscovite (KAl_2_ (FOH)_2_, or (KF)_2_(Al_2_O_3_)_3_(SiO_2_)_6_). Semi-quantitative analysis revealed that quartz is the dominant mineral present in all samples, which dominate about ~ 50–60%, as quantified using *Match! ® (Crystal Impact, Germany)* software. Furthermore, it was also shown the presence of pyrite (~ 15%), which poses threats to the environment and human health. This implies that collected AMT from the artisanal and small-scale mine is classified as hazardous. Due to this, AMT collected from an ASGM plant in Mainit, Davao De Oro, should be reused or recycled to avoid its further harmful effect. Although the negative impact of this waste to the environment are beyond the scope of this study, crucial attention should be focused on this issue. It is also important to note the presence of calcite, which could be due to the use of lime during the smelting process and the innate properties of the host ore^20^. It is also shown that CFA and SA contain significant minerals associated with aluminosilicate, such as muscovite. Some of the minerals detected in AMT were also detected for both samples, such as quartz and calcite. Calcite acts as precursor in the formation of calcium aluminates silicate hydrate (CASH), which consequently enhanced the strength properties of GP.

**Figure 5:**
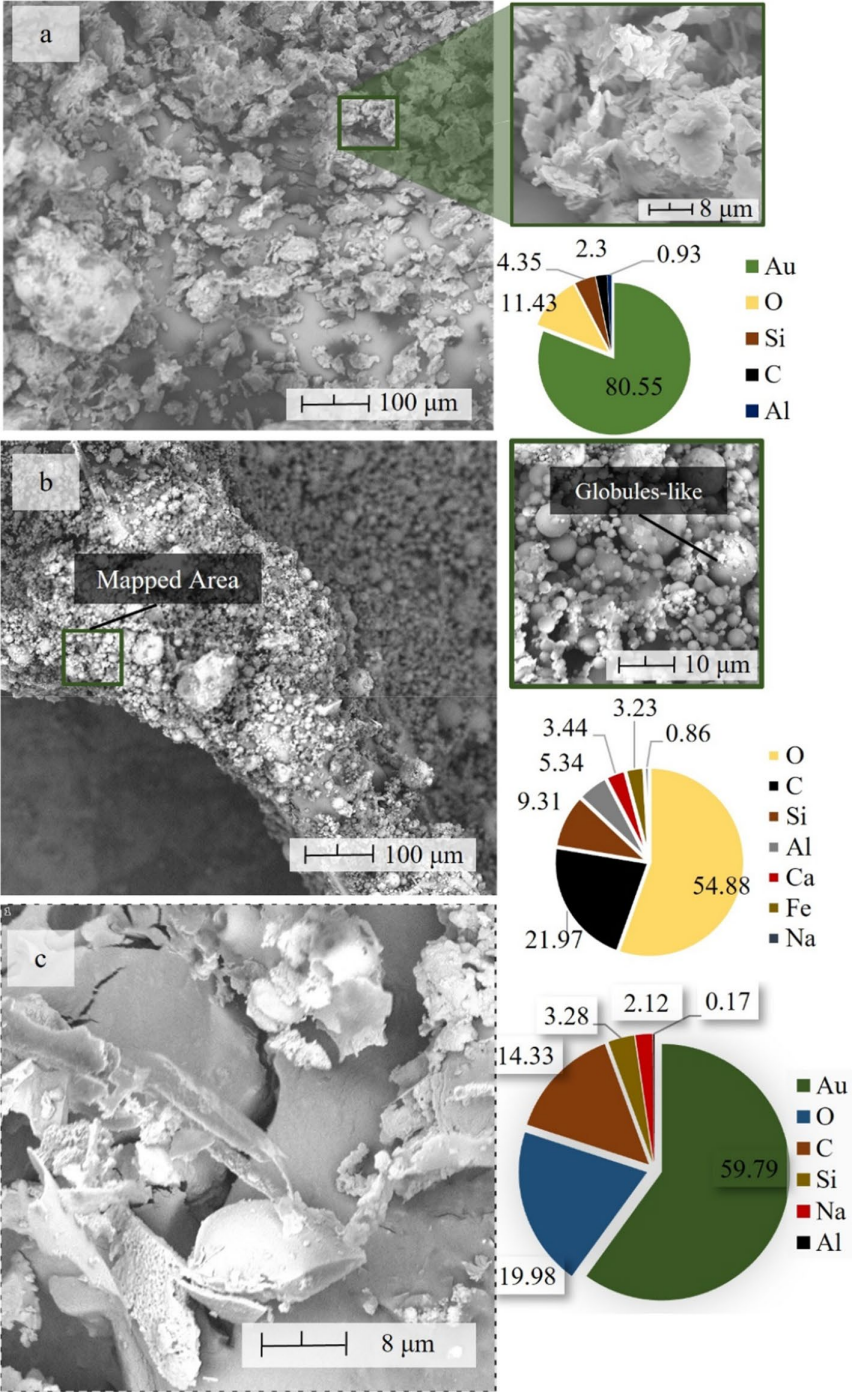
Morphology and elemental point analysis of the industrial waste material (**a**) AMT, (**b**) CFA and the (**c**) solid activator.

AMT exhibited granular-shaped like particles that comprised pluralities of particle sizes, as shown in Fig. 5. Furthermore, it can be observed that in the mapped area with 5000 × magnification, the AMT particles are packed closely with no pore space and exhibits a combination of flaky and flat surface. Elemental point analysis showed the presence of silicon (Si) and aluminum (Al) which occupy 11.43 and 0.93% of the total chemical composition, respectively. Meanwhile, CFA is composed of spherical glass-like particles with non-homogenous sizes, and the synthesized SA shows a flat surface, which characterizes most of its particles. Elemental point analysis also shows that both samples contain a significant amount of Si and Al. These two elements are the primary component to be considered a good material for geopolymerization. This indicates that all raw samples selected for this study are suitable aluminosilicate source materials. It is also important to note that CFA's Si/Al ratio is close to the ideal ratio suggested by Davidovits et al.^7^. EDX result of SA shows the presence of sodium (Na), which is to be expected due to the use of NaOH in the alkali activation process.

### Morphology of the synthesized bismuth oxide nanoparticles

**Figure 6:**
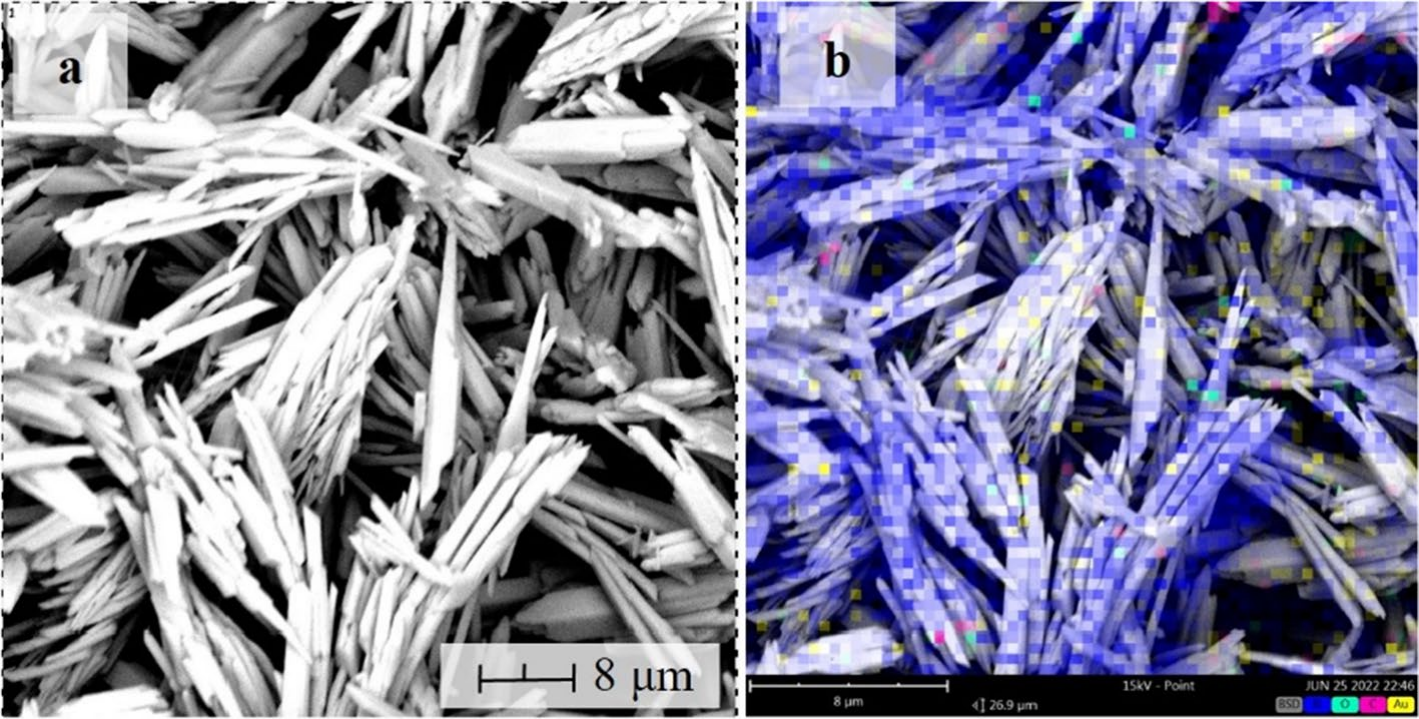
(**a**) Morphology of the synthesized BiNP and its (**b**) elemental map.

SEM photomicrograph of Bi_2_O_3_ NP synthesized via hydrothermal technique at 65 °C for 10 min is shown in Fig. 6. The morphology of the specimen is characterized as nanorods with a minor presence of tabular shape in form. Almost all particles are homogenous, with some agglomerated. Zulkifli et al.^21^ also observed similar SEM results. Mixing Bi(NO_3_)_3_.5H_2_O and Na_2_SO_4_ and dissolution in distilled water causes Bi_2_O(OH)_2_SO_4_ nanomaterial formation. By adding NaOH dropwise, the OH- ions reacted to Bi_2_O(OH)_2_SO_4_ causing the formation of Bi(OH)_3_. Then, this dehydrates and transforms into Bi_2_O_3_ NP during the hydrothermal process. Furthermore, elemental mapping via EDX shows that the synthesized samples comprise Bi, C, and O. The detected carbon is due to carbon tape used during sample preparation. This indicates the purity of Bi_2_O_3_ NP, whereas the detected C is attributed to the carbon tape used for attaching the sample to the holder. Furthermore, particle size analysis of the BiNP shows an average of 150–250 nm.

### Properties of the geopolymer composites

**Figure 7:**
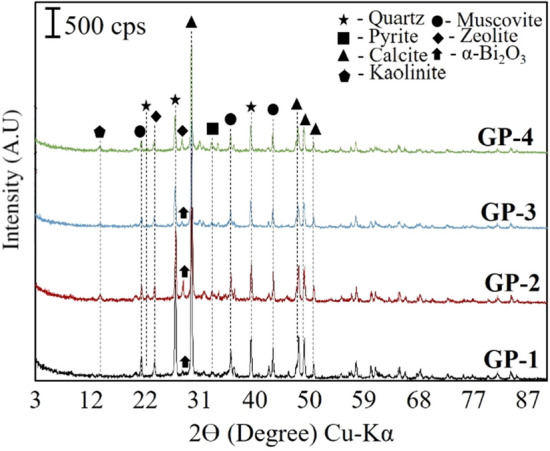
XRD pattern of the geopolymer specimens.

XRD analysis determined the minerals present in the synthesized GP with and without BiNP. As shown in Fig. 7, it can be observed that all GP samples show almost similar mineralogical patterns. Detected minerals are quartz (SiO_2_) (JCPDS card No. 96-900-5021), pyrite (FeS_2_) (JCPDS card No 96-901-5843), calcite (CaCO_3_) (JCPDS card No 96-901-4745), kaolinite (Al_2_Si_2_O_5_(OH)_4_) (JCPDS card No 96-155-0599), muscovite (KAl_2_(FOH)_2_) (96-110-1033), zeolite (Na_2_Al_2_Si_2_O_8_) (JCPDS card No 96-810-1550) and α-Bi_2_O_3_ (JCPDS card No 96-101-0005). Semi-quantitative analysis of the geopolymer samples was determined using *Match! ®(Crystal Impact, Germany)*, which shows quartz as the most predominant mineral present (~ 50%). Calcite (CaCO_3_) is also observed to be high in all samples, which could be attributed to the innate CaCO_3_ present in AMT and SA. Quartz and calcite are insoluble in alkali conditions and do not participate in the geopolymerization process^22^. However, both minerals act as internal fillers during the formation of poly(sialate) framework via oligomerization which could contribute to the strength performance of geopolymer due to their very fine particle size. Moreover, XRD results showed the absence of new phyllosilicates or clay minerals. This implies that some minerals, such as kaolinite, zeolite, and muscovite reformed to their original structure after the dissolution of ions and reorganization of oligomers; and did not produce new clay minerals. Similar results were also observed by the team of Ren et al.^23^ and Opiso et al.^22^ for AMT-based-geopolymer. On the other hand, the sharp peak observed at 2θ around 28°, is attributed to the monoclinic α-Bi_2_O_3_ for GP-1, GP-2, and GP-3 diffraction peak^24^. This indicates that the added BiNP was embedded in the geopolymer matrix. Furthermore, pyrite was also observed to be retained after geopolymerization, which shows that this toxic iron sulfide was immobilized and trapped in the framework.

**Figure 8:**
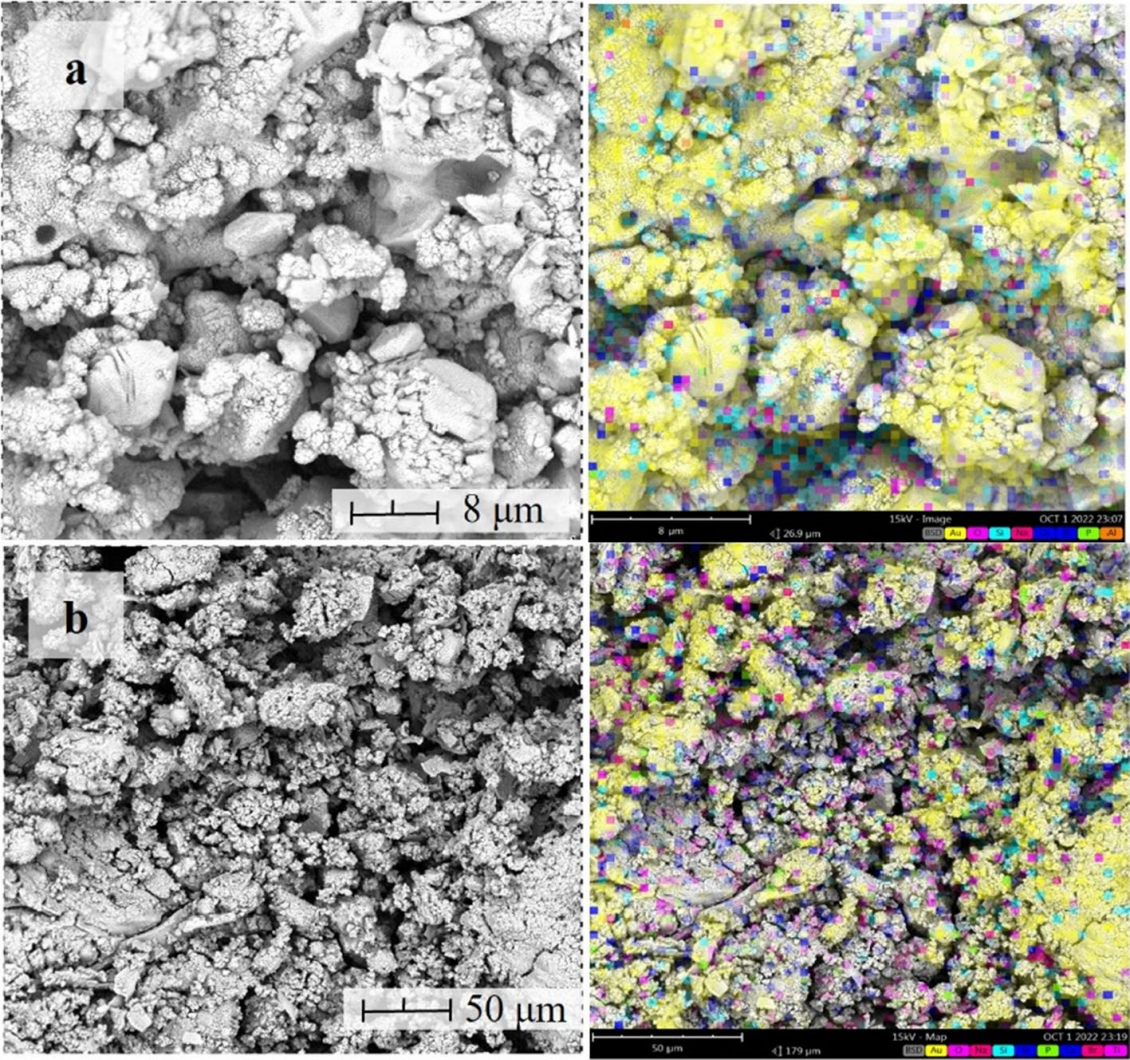
Morphological characteristics and elemental analysis of (**a**) GP-1 and (**b**) GP-2 samples.

**Figure 9:**
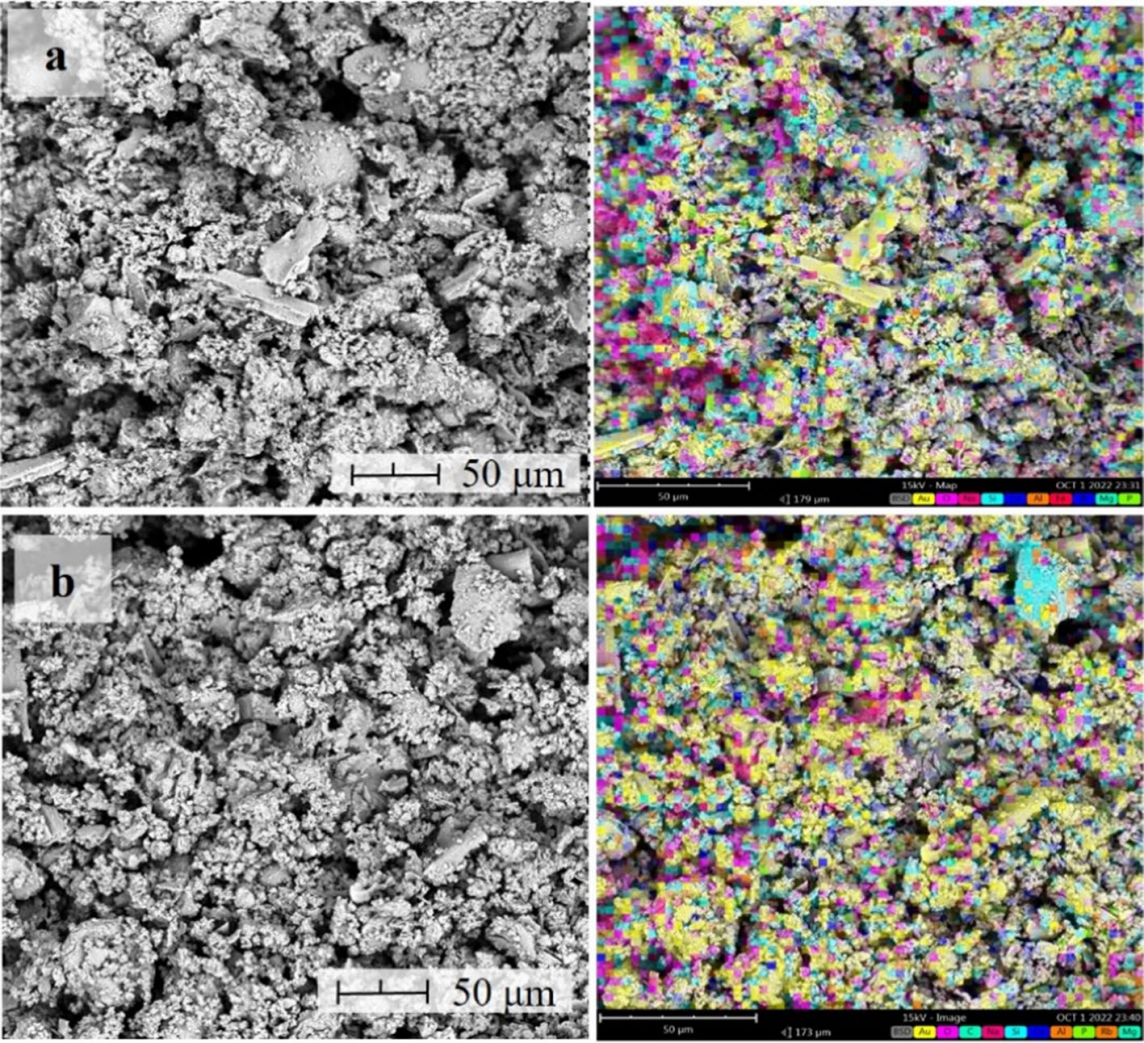
Morphological characteristics and elemental analysis of (**a**) GP-3 and (**b**) GP-4 samples.

Morphological characteristics with corresponding elemental mapping of the synthesized geopolymer samples are shown in Figs. 8 and 9. It can be seen from the photomicrograph that all samples show similar course surface topology, and some asymmetrical shape present in AMT were dissolved after geopolymerization. Furthermore, a gel-like structure was observed for all samples, which could be attributed to the amorphous-to-crystalline minerals formed during the polymerization process. These show similarities with the results of Kiventera et al.^26^ and Opiso et al.^22^ for AMT-based geopolymer. It can also be observed that globules-like particles are incorporated in the matrix of the geopolymers which is attributed to the undissolved CFA. It is also important to note the presence of a few particles with flat surface characteristics observed in the morphology of SA. These indicates that some SA and CFA particle did not participate in the polymerization/polycondensation process and only retain their form. Furthermore, this also indicated that some particles acted only as internal filler. On the other hand, EDX elemental mapping analysis shows that all samples contain silicon (Si) and calcium (Ca), and minor traces of aluminum (Al). This could be attributed to aluminum phyllosilicate minerals formed during the nucleation process. Moreover, it can also be seen from GP-1, GP-2, and GP-3 that bismuth (Bi) was embedded in the geopolymer matrix. It is evident that Bi acted only as an internal filler in the process and did not participate in the geopolymerization process. Furthermore, the detected sodium (Na) for all samples is due to the use of NaOH for alkali activation.

### Compressive strength of the geopolymer composites

**Figure 10:**
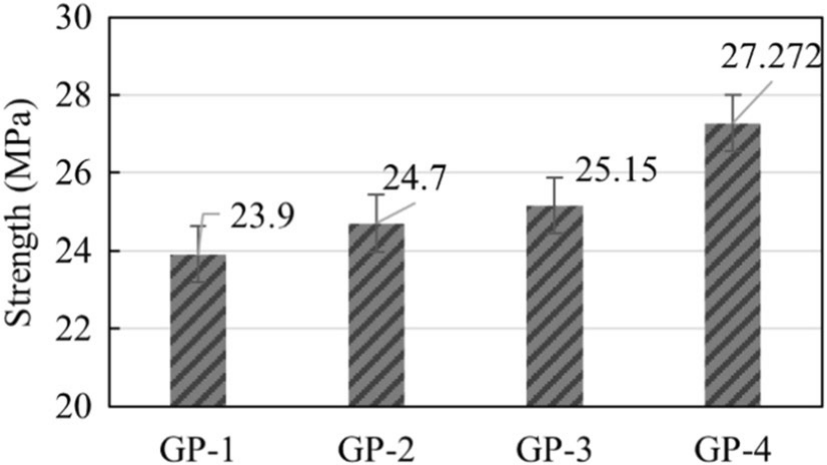
Compressive Strength of the synthesized geopolymer specimens (GP-1 = 15%, GP-2 = 10%, GP-3 = 5% and GP-4 = 0%).

The unconfined compressive strength (UCS) of the synthesized geopolymer samples after 28 days of curing was determined using the universal testing machine. Results show remarkable mechanical properties of all GP samples with average UCS of more than 20 MPa, such that GP-1 is categorized as M_20_ grade, and GP-2, GP-3, and GP-4 are M_25_ grade, as shown in Fig. 10. Additionally, UCS for this study was much higher compared to the studies of Aseniero et al.^18^ and Opiso et al.^22^, despite having the same source of AMT and CFA, indicating that the use of bagasse ash has a relative effect to the enhancement of the strength properties of GP samples. It is also important to note that adding BiNP slightly decreases the geopolymer UCS. The higher the quantity of BiNP added in the matrix, the lower the UCS of GP. This indicates that excessive filler addition results in a decrease in UCS. One possible explanation for this would be that the formation and agglomeration of BiNP within the composite matrix could become the initial crack during UCS testing. Another probable explanation for this is that some BiNP disintegrated during the polycondensation and curing process, which increases the porosity of GP and adversely affects the UCS. Despite this, the value of UCS of all samples is considered significant for various construction applications and comparable to OPC concrete and other GP studies. Moreover, compressive strength at these values can be recommended for small-scale and large residential building.

### Thermogravimetric analysis of the geopolymer composites

**Figure 11:**
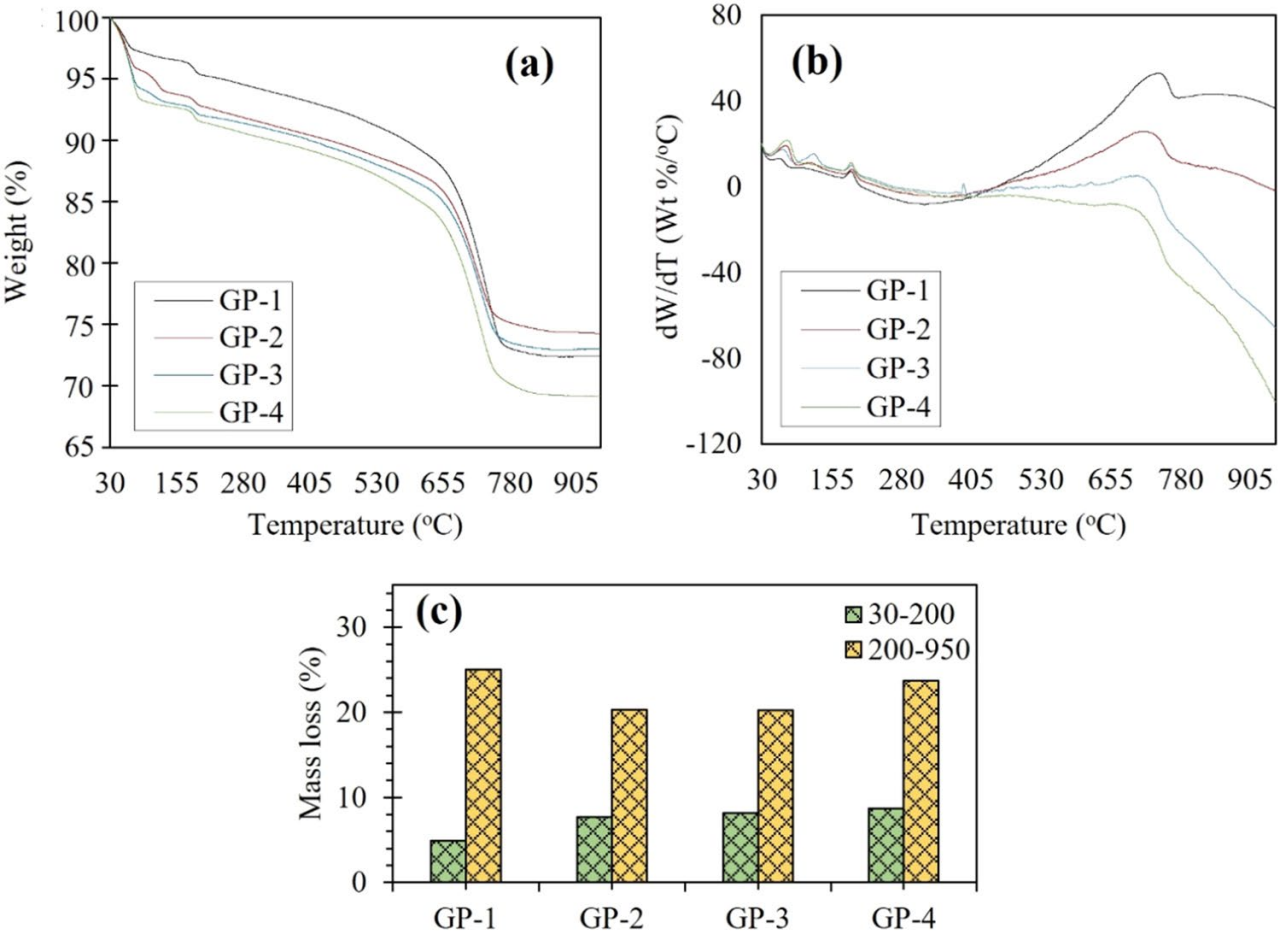
(**a**) TGA and (**b**) DTG curves of the Geopolymer composites in nitrogen atmosphere; (**c**) comparison of mass loss between 30–200 and 200–950 °C.

Throughout its service life, the shielding materials might be exposed to high-to-extreme temperature conditions environment, therefore it is also imperative to investigate the thermal stability of the synthesized geopolymer composites. The thermogravimetric–differential thermogravimetric (TG-DTG) with thermal analysis (TGA) of the geopolymer samples was performed to evaluate its thermal degradation properties. Figure 11 shows the TGA and DTG curves of the specimens.

The TGA spectra of the specimens highlight two significant weight losses: the first in the region from 30 to 200 °C, which is attributed to the dehydration of absorbed and unbounded H_2_O, physically and chemically bonded water embedded within the geopolymer matrix^26,27^. The water release in this region due to evaporation is approximately 4–10% of the total mass loss. Water plays a crucial role in the geopolymerization process since it acts as the medium to dissolve aluminosilicate into aluminum and silica monomers and participate in polycondensation and polymerization. Water is continuously released during the curing process to form an amorphous phase and covalently bonded three-dimensional network of polymeric chain^26^. It is also important to note the degradation observed around 175 °C for all samples, which is due to the dehydration of calcium aluminate silicate hydrate (CASH)^28^. The second one is between 200 and 800 °C, which is due to the release of bounded H_2_O from Si–OH or/and Al–OH functional groups via condensation/polymerization^29^. The degradation at this region could also be assigned to the decomposition of carbonate compounds and dihydroxylation of the OH groups. The highest mass loss was observed from 600 to 800 °C due to the de-carbonation of CaCO_3_. Similar weight loss was also observed by other studies^30,31^. In general, the addition of BiNP to the geopolymer matrix has shown a significant effect on the thermal stability, with each mix varying the amount of added filler. GP-4 exhibited the highest weight loss (~ 32%) followed by GP-1 (~ 30%), GP-2 (~ 29%) and GP-3 (~ 28%). The weight loss of all geopolymer samples was stabilized after 850 °C. Moreover, Fig. 10 also shows that majority of the degraded samples were in the 200–800 °C range. Evident weight losses at 10% and 30% in the TGA are presented in Table 2.

**Table 2:** Thermal stability of the geopolymer composites obtained from TGA.

Sample	10% weight loss (°C)	30% weight loss (°C)	Residue (%)
GP-1	60.88	728.39	72.018
GP-2	60.45	713.89	70.031
GP-3	51.76	723.70	71.623
GP-4	49.31	722.90	66.569

### Dielectric properties of geopolymer composites

**Figure 12:**
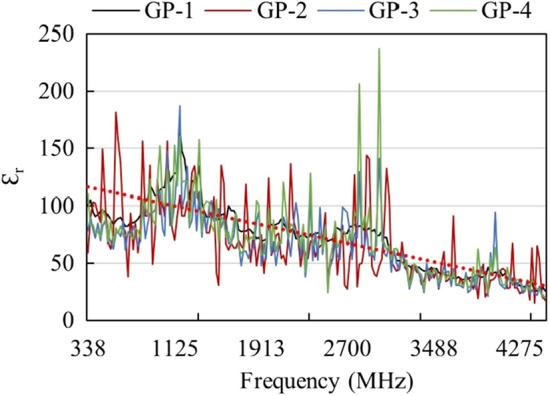
Dielectric properties of the synthesized geopolymer composites.

The permeability or dielectric properties are important parameters in determining the polarization and charge localization effect of shielding material. In this study, the dielectric constant of all geopolymer composite samples has been measured as a function of frequency to understand the effect of the added BiNP filler on the polarization characteristics. Figure 12 shows the measured dielectric constant. It can be observed that the dielectric constant for all samples decreases with increasing frequency. These findings corroborate the works of Chuewangkam et al.^32^ and Hanjitsuwan et al.^33^, which could be attributed to the highly porous microstructure characteristics of the geopolymer composites. In general, there are no significant differences between the permeability of the synthesized geopolymer composites indicating that BiNP did not alter the charge localization. Dielectric properties are a function of the polarization of the material. At low frequencies, the dielectric response is due to the interfacial polarization of the composite material. The molecules obtained sufficient time at this frequency to rotate and change orientation towards the applied alternating current^32^. However, high frequency causes relaxation of the polarization process due to the insufficient time for re-orientation, thereby decreasing the Ɛ_r_^34^.

### EMI shielding effectiveness and mechanism of geopolymer composites

**Figure 13:**
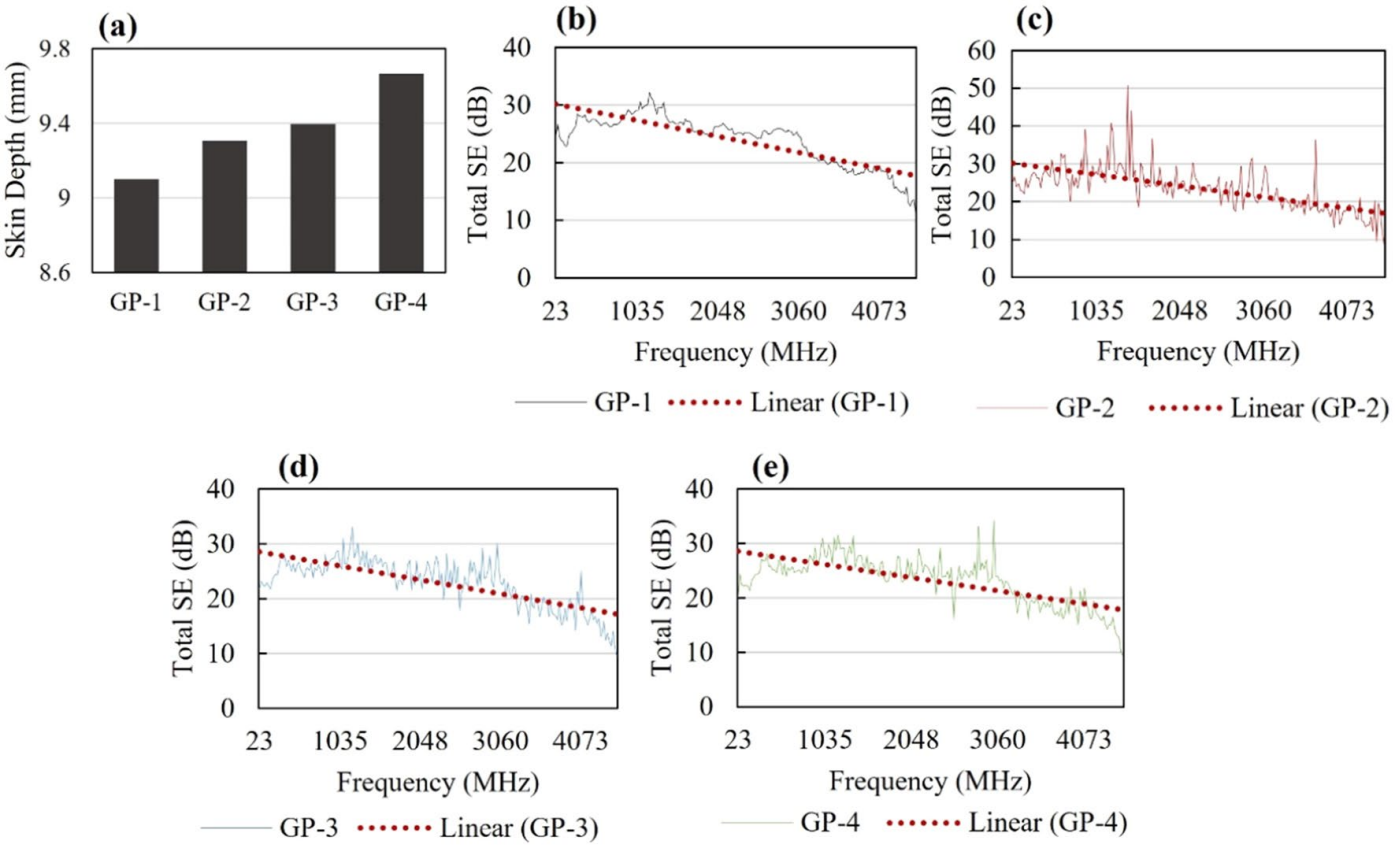
Total EMI-SE of geopolymer samples with 10 mm thickness (**a**) GP-1, (**b**) GP-2, (**c**) GP-3 and (**d**) GP-4.

The concepts of EMI-SE primarily lie in the reflection, absorption, and multiple reflections of EM waves through a shielding material. EMI reflection occurs at the border between any two media with significant discrepancies in their electric and magnetic impedances and is highly dependent on the conductivity of the material. The higher the material's conductivity, the more suitable it is to reflect EM waves. On the other hand, EMI absorption tends on the magnetic component of the EM wave and is mainly dependent on the thickness of the materials, including its conductive properties. The amount of absorption that a material could have been approximated in by the factor ${e}^{-\frac{t}{\delta }}$ , where $t$ is the thickness of the material while $\delta$, is the skin depth. $\delta$ is defined in the equation below

7 $$\delta =\frac{1}{\sqrt{\pi f\mu {\sigma }^{2}}}$$

where $f$ is the frequency, $\mu$ is the permeability of the material and $\sigma$ is the conductivity. Given that the thickness of all synthesized geopolymer composites were fixed at 10 mm, the computed $\delta$ for all synthesized geopolymer composites are shown in Fig. 13a. Furthermore, part of the mechanism of EMI absorption is weakening the incident EM waves through eddy currents. This eddy current generates a magnetic field that opposes the external magnetic field. Shielding material that has high electrical conductivity properties creates a stronger eddy current. Multiple reflections operate via internal reflection within the material, resulting in the scattering of EM waves. This is observed in composite materials with large interfacial areas with porous structures^4^.

The results of the EMI shielding effectiveness of the GP specimen incorporated with bismuth oxide nanoparticles (BiNP) with variation of % weight content are illustrated in this section. SE_R_ (contribution by reflection) and SE_A_ (contribution by absorption) were evaluated from the obtained S (S_11_ or S_22_ and S_12_ or S_21_) parameters as well as the reflection (R) and transmission (T) coefficient. For this study, SE_MR_ (contribution by multiple reflections) can be neglected since ${SE}_{T}>10 dB$^35^ and the skin depths are less than the samples thickness therefore, Eq. (6) can be simplified as,

8 $${SE}_{Total}\left(dB\right)={SE}_{R}+{SE}_{A}$$

The shielding efficiency tested in the frequency range 20 MHz < f < 4500 MHz. These ranges are significant for commercial applications such as signals emitted from TV, mobile phones, wireless LAN, and radar image.

As can be seen in Fig. 13, as the frequency of the EM wave increases, the trend of the shielding efficiency of the GP samples decreases. This indicates that attenuating properties of the samples weaken at higher EM waves. The average total EMI-SE of GP-4 (neat GP) was 21.2 dB and slightly increased up to 23.6 dB (GP-3), 22.9 dB (GP-2), and 22.1 dB (GP-1) with the introduction of 5, 10, and 15% BiNP, respectively. This means that the addition of hydrothermally synthesized BiNP improves its EMI-SE characteristics; however, it only increases the efficiency by approximately 4–10%. It can also be noted that EMI-SE of GP was shown to improve more when 5% of BiNP was introduced and was observed to decrease when the % content was increased to 15%. This denotes that residual BiNP in the matrix of the geopolymer negatively affects the shielding efficiency of GP. This result also implies that 5% of BiNP is the ideal ratio for GP mixture with optimum properties.

**Figure 14:**
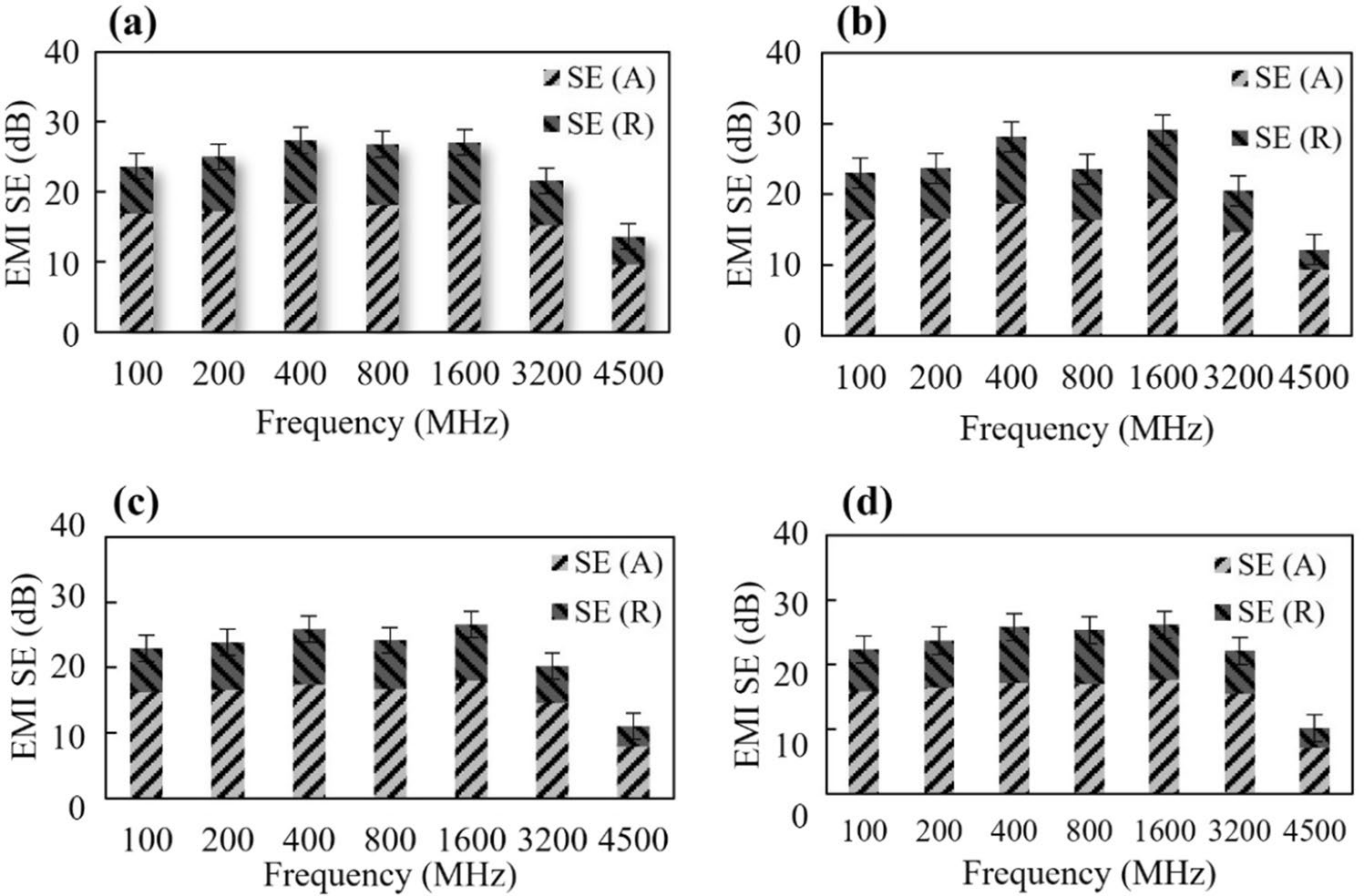
Distribution of total EMI-SE (**a**) GP-1, (**b**) GP-2, (**c**) GP-3 and (**d**) GP-4.

Table 3 compares the EMI shielding performance of GP-3 to other published attenuation properties of cement and geopolymer composites with added filler. In general, EMI SE can be improved by increasing the thickness of the shielding material. Therefore, it is imperative to normalize total SE by sample thickness (SEtotal/d)^36^. It is evident that GP-3 (with 5% BiNP) is comparable and even outperformed other GP and cement composites when sample thickness is considered. Furthermore, despite the insufficient evidence of BiNP as high EMI SE material, GP-3 possessed 20 × the attenuating properties compared to normal concrete^5^ and 2 × to the stainless-steel dust-added cement^13^. These results indicated that 5% of BiNP used as additive filler to GP is a competitive structural material with significant EMI-SE properties. According to Eqs. (10)–(11), SE_R_ (contribution by reflection) and SE_A_ (contribution by absorption) are plotted in Fig. 14. In general, SE_A_ was higher than SE_R_ for all geopolymer samples, indicating that the absorption coefficient was the primary EMI shielding mechanism.

**Table 3:** Comparison of EMI SE of cement and GP with added filler to the current study.

Sample	Filler	Thickness (mm)	Frequency (GHz)	Total SE (dB)	References
Fly ash-bsed GP	3.75% pyrolyzed cork	7	8.2–12.4	15.9	Novais et al. (2019)^34^
Metakaolin-based GP	5% (SiO_2_)-coated carbon nanotubes	2	8.2–12.4	24.2	Zhu et al. (2022)^9^
Cement	Stainless-steel dust	5	0.5–1.5	6–9	Fan et al. (2017)^13^
Cement	1.0% Carbon fiber	4.2	1.5	13	Wen and Chung 2007^35^
Normal concrete	–	10	0.3–1.5	1.5–9.1	Lee et al. (2021)^5^
AMT-based GP	5% BiNP	10	4.5 GHz	23.5	This work

## Conclusion

This study was focused on investigating the compatibility of utilizing industrial waste-based geopolymer composite in EMI-shielding applications with high thermal properties. Furthermore, to enhance the EMI shielding capabilities of such material, a hydrothermal synthesized BiNP was used as an additive filler in the matrix with different aspect ratios and was tested for various characteristics. Based on the results obtained, the following conclusion were drawn:

1. Morphological and Elemental analysis showed that the BiNP has been distributed in the matrix of the GP samples.
2. Unconfined Compressive Strength of synthesized GP samples shown to exceed 20 MPa. However, the addition of BiNP has shown to decrease its mechanical properties.
3. Increasing the content of BiNP added in the matrix of the GP samples showed a gradual increase in the EMI Shielding in the frequency range from 20 to 4500 MHz. However, the increase was observed to be only 4–10%.
4. The shielding efficiency test of the GP samples also shows that the absorption coefficient was the primary EMI shielding mechanism.
5. Thermogravimetric Analysis shows that the addition of BiNP significantly improves the thermal stability of the GP samples.
6. Based on the UCS, EMI-SE, and Thermal property results, the synthesized GP samples incorporated with BiNP can be recommended for small-scale construction and small residential building.

## Data Availability

All data analyzed during this study are included in this published article. The datasets used and/or analyzed during the current study available from the corresponding author on reasonable request.
